# Pretreatment Circulating HPV16 DNA Viral Load Predicts Risk of Distant Metastasis in Patients with HPV16-Positive Oropharyngeal Cancer

**DOI:** 10.3390/cancers16061163

**Published:** 2024-03-15

**Authors:** Agnieszka Maria Mazurek, Iwona Jabłońska, Marek Kentnowski, Urszula Kacorzyk, Mirosław Śnietura, Tomasz Wojciech Rutkowski

**Affiliations:** 1 Center for Translational Research and Molecular Biology of Cancer, Maria Sklodowska-Curie National Research Institute of Oncology Gliwice Branch, Wybrzeze Armii Krajowej 15, 44-102 Gliwice, Poland; 2 I Radiation and Clinical Oncology Department, Maria Sklodowska-Curie National Research Institute of Oncology Gliwice Branch, Wybrzeze Armii Krajowej 15, 44-102 Gliwice, Poland; 3 Department of Pathomorphology and Molecular Diagnostics, Medical University of Silesia in Katowice, Medyków 18, 40-752 Katowice, Poland; 4 Radiotherapy Department, Maria Sklodowska-Curie National Research Institute of Oncology Gliwice Branch, Wybrzeze Armii Krajowej 15, 44-102 Gliwice, Poland

**Keywords:** head and neck cancer, oropharyngeal cancer, chemoradiotherapy, human papillomavirus, distant metastasis, circulating tumor-related HPV16 DNA, plasma, viral load

## Abstract

**Simple Summary:**

Convincing data have indicated that human papillomavirus (HPV)-related oropharyngeal squamous cell carcinoma (OPSCC) is associated with much better locoregional control and survival than its HPV-unrelated counterparts. It is also worth noting that the distant metastases (DM) rate does not differ between HPV-related and HPV-unrelated OPSCC. Recently, the analysis of HPV in liquid biopsy (circulating tumor-related HPV DNA, ctHPV) has gained significant prognostic importance. Little is known about the importance of viral load (VL) levels of ctHPV16 in OPSCC on survival. Meanwhile, our results provide comprehensive data that the pretreatment ctHPV16 VL in liquid biopsy may be a biomarker of the risk of DM.

**Abstract:**

Background: There are definite reasons to implement molecular diagnostics based on the measurement of human papillomavirus (HPV) DNA in liquid biopsy into clinical practice. It is a quick, repeatable, and health-safe test for cancer biomarkers in the blood. In this study, we investigated whether the circulating tumor-related HPV16 (ctHPV16) viral load (VL) in patients with oropharyngeal squamous cell carcinoma (OPSCC) was important for determining the risk of locoregional recurrence-free survival (LRFS), metastasis-free survival (MFS), and overall survival (OS). Methods: This study included 91 patients with ctHPV16-positive OPSCC who had been treated with radical radiotherapy and chemotherapy. The VL was measured using quantitative PCR (qPCR) and a probe specific for HPV16. Based on 10 years of follow-up, the 2-, 3-, 5-, and 9-year LRFS, MFS, and OS were estimated. Results: The 5-year actuarial LRFS, MFS, and OS rates of patients with ctHPV16-positive/OPSCC were 88%, 90%, and 81%, respectively. The VL was significantly higher in patients who subsequently developed distant metastases (DM) than in those who did not (VL 4.09 vs. 3.25; *p* = 0.009). In a Cox proportional hazards regression model for MFS, a higher ctHPV16 VL appeared to be a significant independent prognostic factor for the occurrence of DM (HR 2.22, *p* = 0.015). The ROC curve revealed a cutoff value of 3.556 for VL (*p* = 0.00001). Conclusions: A high VL before treatment indicates patients with a significant risk of DM, and should be used in OPSCC treatment stratification.

## 1. Introduction

Head and neck cancer (HNC) is the seventh most common cancer globally, accounting for an estimated 890,000 new cases (4.5% of all cancer diagnoses around the world) and 450,000 deaths per year (4.6% of global cancer deaths) [1]. The overall 5-year survival rate for HNC patients is 58%, with the highest survival rate being 63% for OPSCC [2]. However, among OPSCC, the five-year overall survival rates in HPV-positive patients are considerably higher than in HPV-negative patients [3]. The current radical treatment of OPSCC involves surgery, radiotherapy (RT), or chemoradiotherapy (CHRT). RT or CHRT can supplement the surgical approach or be used as the sole treatment. Some intensification strategies, including PF-based (cisplatin and 5-fluorouracil) or TPF-based (docetaxel, cisplatin, and 5-fluorouracil) induction chemotherapy (iCHT), have been explored, but the advantages of iCHT over CHRT have not been confirmed [4].

A new light on risk stratification has emerged with the confirmation of high-risk HPV as an etiological factor of OPSCC and the introduction of a unique classification of lymph node disease in this group [5]. Patients with HPV-related OPSCC usually present with a more advanced nodal stage but still have a better prognosis. Although there is strong evidence showing that HPV-related OPSCC is associated with significantly better locoregional control and survival than its HPV-unrelated counterparts [6,7], the risk of locoregional treatment failure in patients with HPV-related cancer remains high (25%) [8,9]. It is also worth noting that the distant metastases (DM) rate does not differ between HPV-related and HPV-unrelated OPSCC [9].

Recently, the analysis of HPV in liquid biopsy (circulating tumor-related HPV DNA, ctHPV) has gained significant prognostic importance, both in monitoring the effectiveness of treatment and for follow-up [10,11,12]. In addition, high sensitivity (81%) and specificity (95–98%) of ctHPV have been demonstrated in pretreatment blood samples [11,13]. However, there are no accurate survival analyses that consider the level of ctHPV copies in the blood before starting treatment. In this article, we consider whether quantification of pretreatment circulating tumor-related HPV type 16 (ctHPV16), defined as the viral load (VL) (number of detected copies per milliliter of plasma), will determine the risk of locoregional failure or distant spread.

## 2. Materials and Methods

### 2.1. Patient Population and Treatment

This study analyzed the VL in patients prior to treatment; therefore, those who had undergone a radical surgical approach, tonsillectomy, lymphedectomy, or tumor removal before treatment were excluded from the study. Tumor staging was performed using the following: 18F-fluorodeoxyglucose positron emission tomography-computed tomography (18F-FDG PET-CT) in 48 patients (53%), magnetic resonance imaging (MRI) in 19 patients (21%), or computed tomography (CT) in 24 patients (26%). All patients underwent radical treatment with RT alone (*n* = 7), CHRT (*n* = 22), or iCHT (*n* = 62), followed by RT (*n* = 16), or CHRT (*n* = 46) at the Maria Sklodowska-Curie National Research Institute of Oncology Gliwice Branch between 2012 and 2020. RT included 70 Gy in 35 fractions given in 7 weeks (2.0 Gy/fraction) or 70.2 Gy in 39 fractions given in 5.5 weeks (1.8 Gy/fraction) to the gross tumor volume. CHRT included RT and cisplatin administered at a dose of 100 mg/m^2^ on days 1, 22, and 43. Clinical target volume included volumes of potential microscopic spread of disease and was irradiated to 50 Gy in 25 fractions (2.0 Gy/fraction) or 54 Gy in 30 fractions (1.8 Gy/fraction). iCHT consisted of 2–3 cycles of TPF (docetaxel 75 mg/m^2^, cisplatin 75 mg/m^2^, d1 and 5-fluorouracil 750 mg/m^2^ d1–d4) or PF (cisplatin 100 mg/m^2^, d1 and 5-fluorouracil 1000 mg/m^2^ d1–d5). The tumor status was defined according to the American Joint Committee on Cancer (AJCC, 8th edition). Treatment response was evaluated according to the criteria for assessing response in solid tumors (RECIST) [14].

### 2.2. Follow-Up of Patients after Completion of Treatment

We followed patients with the schedule as below: All patients were monitored periodically: the first visit was approximately 12 weeks after the end of treatment, then every 3 months for the first year, and then every 6 months or more often if necessary. At week 12 after treatment, MRI, CT, or 18F-FDG PET-CT was usually performed. Thereafter, laryngological examination supplemented with endoscopy during each follow-up visit was performed. Subsequent radiological examination (CT or MRI) was performed after another 6 months (one year after treatment completion) and then every year. We also performed a chest X-ray every 2 years. When we suspected local or nodal recurrence we performed MRI or CT including head and neck regions; additionally, a chest X-ray and abdominal ultrasound. If dissemination was suspected or the presence of a mass that could not be more precisely determined by CT or MRI, a 18F-FDG PET-CT scan was additionally performed. In this cohort of patients, ctHPV DNA recurrences in liquid biopsy was an example of recurrence suspicion and in such cases 18F-FDG PET-CT was also recommended. So due to ctHPV DNA recurrence, 18F-FDG PET-CT that included the body to the line above the knee was performed. Whole-body imaging protocols were always selected when distant metastases were suspected based on abdominal ultrasound, X-ray, elevated tumor markers, and the presence of ctHPV. The median follow-up time for LRFS, MFS, and OS was 45.00 months (range 0.23–112.27, l.q −30.67, u.q. 63.50), 45.00 months (range 0.23–112.27, l.q. 29.50, u.q. 65.27), and 50.03 months (range 1.77–113.90, l.q 35.33, u.q. 70.70), respectively. This project was approved by the Bioethics Committee of the Maria Sklodowska-Curie National Research Institute of Oncology Gliwice Branch, Poland. Informed consent was obtained from all subjects involved in the study.

### 2.3. Methodology for Determining ctHPV16 in Blood

Plasma samples collected from the date of diagnosis to initiation of therapy were considered “pretreatment”. ctHPV16 testing was performed immediately after venous blood collection, plasma separation, and DNA isolation. Peripheral blood was separated by double centrifugation immediately after blood donation (10 min at 4 °C, 300 g, and 1000 g). DNA was extracted from 1 mL of plasma by the Genomic Mini AX Body Fluids kit (A&A Biotechnology, Gdynia, Poland). The remaining plasma was preserved at −80 °C and used for repetitions.

For ctHPV16 testing and VL quantification, we used qPCR based on TaqMan technology (probes and starters), along with co-amplification of TERT (human telomerase reverse transcriptase) as a marker of the total circulating cell-free DNA (cfDNA) present in the samples. Each measurement consisted of a standard curve (plasmid construct), negative control, and sample. All PCRs were performed using a Bio-Rad CFX96 real-time system (Bio-Rad Laboratories, Hemel Hempstead, UK). The standard curve was used for determination of the number of copies in each sample using the following formula: CN = SQ × V_eluate_/V_plasma_), where CN is the copy number of ctHPV16 DNA per mL (copies/mL), SQ is the copy number determined using the standard curve for HPV16/PCR using the CFX Maestro Version: 5.3.022.1030. Software (number of copies), V_eluate_ is the total volume of DNA (µL) obtained after extraction, and V_plasma_ is the volume of plasma used for DNA extraction (mL) [15]. ctHPV16 VL in plasma was expressed as log_10_ of VL (log_10_ of copy number of HPV16 DNA per 1 mL) for all statistics. In this study, the term circulating tumor-related HPV16 DNA (ctHPV16) referred to HPV16 DNA detected in the total cfDNA isolated from plasma samples.

### 2.4. Strategy for Determining HPV Status in This Study

From 2012 to 2017, 722 HNC patients were examined; ctHPV16 was detected in 116 (16.07%). In the following years, 2018–2020, the test was performed only in patients with OPSCC; 427 patients with OPSCC were examined, and ctHPV16 was detected in 98 (22.95%). A total of 214 ctHPV16-positive OPSCCs were collected from 2012 to 2020. To study the prognostic significance of VL, only those patients who underwent RT or CHRT were included. Between 2012 and 2020 there were 91 patients, both with histologically confirmed OPSCC and positive for ctHPV16. There were 71 (78%) tonsil cancers, 19 (21%) on the base of the tongue, and 1 (1%) cancer of the palate. Out of 91 cases, 77 had the presence of HPV confirmed by PCR or a test for p16 was performed. There were 38 cases confirmed both by p16 and PCR; 32 confirmed only by PCR; and 7 confirmed only by p16. In 14 cases, tumor tissue was not available. The limitation of the tissue testing was the use of archival tumor material. The sensitivity of the test was estimated at 72% and the specificity at 100% [15].

### 2.5. Statistical Analysis

Statistical analyses were performed using Statistica Software, version 13 (TIBCO Software Inc., Palo Alto, CA, USA) and Set Plus version 5.0.96. (StatSoft Polska Sp. z o.o., Kraków, Poland). Age categorization was performed according to the median values. Cigarette consumption categorization included non-smoker or smoker status, with smoking status assigned to current and former smokers. For statistical analysis, T3 or T4 tumors were assigned to one category and T1 or T2 tumors were assigned to the second category. For N classification, N3 status was assigned to one category and N0–2 status to the second category. Continuous data were shown as median values with interquartile ranges (25–75%, IQR 25–75) and mean values with standard deviation. Normality of distribution was tested using the Shapiro–Wilk test. Levene’s test was used to determine the homogeneity of the variances. After confirming the homogeneity of variances, a *t*-test (two-sided *p*-value) was performed to compare the VL. Multiple linear regression was used to evaluate the predictor(s) of VL level, standardized beta coefficients (b*), and *p*-values. Statistical significance was set at *p* < 0.05. Locoregional recurrence-free survival (LRFS) was defined as the length of time after treatment ended, when the patient survived without symptoms of regional or lymph node recurrence. Metastasis-free survival (MFS) was defined as the length of time from the start of treatment for cancer when a patient was still alive and had no evidence of distant metastases. The overall survival (OS) rate was defined as the percentage of individuals who survived after treatment initiation. Cox proportional hazards models were used to estimate the hazard ratios (HRs) for LRFS, MFS, and OS. To estimate the predictor(s) of LRFS, MFS, and OS in the full model, multivariable regression analysis was performed (all effects), and backward elimination was used to find the best predictor. STATISTICA performed a backward search for the best subset of predictors by building a full model (including all defined predictors) and then sequentially removing variables according to the Wald best improvement criterion. The *p* value used for elimination was above 0.15. Test power analyses were performed for the primary outcomes. The receiver operating characteristic (ROC) curve was used to assess the overall diagnostic performance of the ctHPV16 test for DM prediction and to select the optimal cutoff value and area-under-the-curve (AUC) calculations.

## 3. Results

### 3.1. Patients’ Characteristics

The median age of the patients was 59 years (range: 30–80 years). The study included 59 men (65%) and 32 women (35%). The following distribution was for the T classification: T1—3 (3%), T2—36 (40%), T3—28 (31%) and T4—24 (26%). The distribution of the N classification was N0–2 (2%), N1–46 (51%), N2–18 (20%), and N3–25 (27%). There were 40 (44%) smokers and 51 (56%) non-smokers in this group. Four patients developed a second neoplasm in follow-up: one in the hypopharynx, one in the pancreas, and two in the skin.

### 3.2. Pretreatment VL of ctHPV16 in Relation to Clinical Parameters

**Figure 1:**
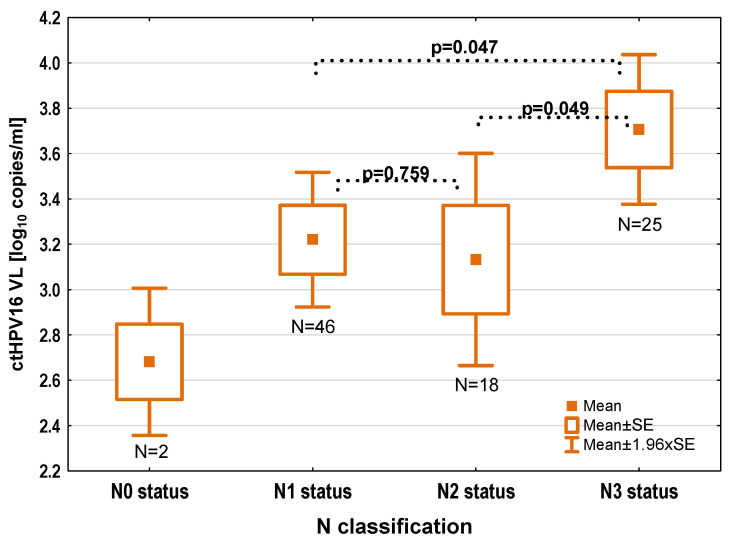
ctHPV16 VL level depending on N classification.

The median viral copy number was 2367 copies/mL, with a range between 32 and 976,145, a lower quartile of (l.q.) 424, and an upper quartile of (l.q.) 11,580. A Shapiro–Wilk test revealed that the distribution of the values was not normal (*p* < 0.0001). After converting the values to log_10_, a normal distribution was obtained (*p* = 0.159). Therefore, all statistical analyses were performed on the log_10_ of ctHPV16 DNA copy number per mL, which defined the VL used in this study. The following VL values were obtained: median 3.37, mean 3.32, lower quartile 2.63, upper quartile, 4.06; minimum, 1.5; and maximum, 5.55. The VLs and *p*-values (*t*-tests) according to clinical parameters are presented in Table 1. Univariate analysis revealed that patients with N3 status had significantly higher VL than patients with N0–2 status (mean ± SD: 3.71 ± 0.84 (*n* = 25) vs. 3.18 ± 1.01 (*n* = 66), *p* = 0.022). A more detailed analysis showed that patients with N3 status had a significantly higher VL (*n* = 25, mean VL 3.71) than patients with N1 status (*n* = 46, mean VL 3.22) (*p* = 0.047) or N2 status (*n* = 18, mean VL 3.13) (*p* = 0.049) (Figure 1).

A significant positive correlation between VL and N status (N1 N2 N3) was observed (*p* = 0.043; r = 0.212; r^2^ = 0.045). There was no correlation between the T status and VL (*p* = 0.181; r = 0.141; r^2^ = 0.020).

Multiple regression analysis was performed to determine the factors affecting VL. The following variables were included in the model: T3/T4 status vs. T1/T2, N3 status vs. N0–2, sex, cigarette consumption, and age < 59 vs. age ≥ 59 years. The multiple regression model (*p* < 0.017) showed that N3 status (*p* = 0.052, beta coefficient = 0.204) and male sex (*p* = 0.092, beta coefficient = 0.177) were predictors of higher VL of ctHPV16 in the blood.

### 3.3. Parameters Influencing OS, LRFS, and MFS in ctHPV16-Related OPSCC Patients

**Figure 2:**
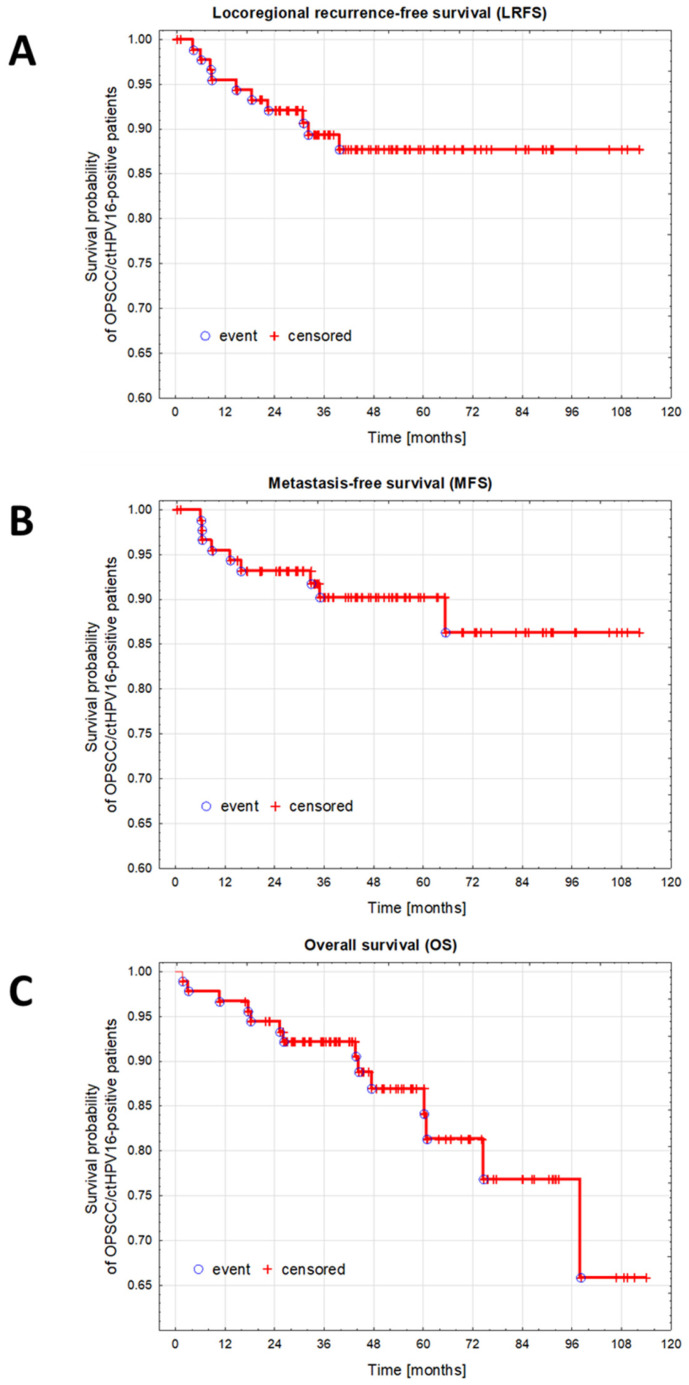
Kaplan–Meyer survival analyzes of patients with ctHPV16-positive OPSCC. (**A**). Survival probability plot for LRFS. The median follow-up time for LRFS: for censored *n* = 81, median 49.00 months (range 0.23–112.27); for event *n* = 10, median 16.58 months (range 4.17–39.70). (**B**). Survival probability plot for MFS. The median follow-up time for MFS: for censored *n* = 82, median 48.48 months (range 0.23–112.27); for event *n* = 9, median 13.10 months (range 6.07–65.27). (**C**). Survival probability plot for OS. The median follow-up time for OS: for censored *n* = 77, median 50.43 months (range 16.87–113.90); for event *n* = 14, median 34.83 months (range 1.77–97.97). Abbreviations: VL—viral load, ctHPV16, circulating tumor-related human papillomavirus type 16; OPSCC, oropharyngeal squamous cell carcinoma.

The 2-, 3-, 5-, and 9-year actuarial OS, LRFS, and MFS rates (the survival rate—the percentage of people in a study who are alive) are presented in Table 2. Figure 2 shows the detailed LRFS and MFS rates of ctHPV16-positive OPSCC patients.

The percent of local or nodal recurrence up to month 16 was 50% (5/10); LRFS in Figure 2A. However, the number of DM events up to month 16 was 67% (6/9); MFS in Figure 2B.

Cox proportional hazards models were used for HR calculation for the following parameters: T3/T4 vs. T1/T2, N3 vs. N0–2, sex, cigarette consumption, age < 59 years vs. age ≥ 59 years, and VL. Table 3 presents the multivariate Cox proportional hazards regression models for LRFS, MFS, and OS of the patients with ctHPV16-related OPSCC treated with RT/CHRT. The results of backward elimination revealed that patients with a higher VL presented a significantly shorter MFS (HR 2.22, *p* = 0.015; Table 3). For tonsil cancer only (*n* = 71), Cox proportional hazards models were used for HR calculation among the same parameters. The results of backward elimination proved VL as an independent factor for DM. Patients with a higher VL presented a significantly shorter MFS (HR 2.43, *p* = 0.016).

We did not identify independent prognostic factors for OS in this group; however, for LRFS, advanced T status (T3–T4), although not statistically significant, had an impact on locoregional recurrence (HR 7.27, *p* = 0.060, Table 3). All locoregional recurrences appeared only in the patients with a primary tumor localized in the tonsil.

### 3.4. High VL as a Predictor of DM

All DM occurred in the patients with primary tumor localization in the tonsil (9/9). In eight cases (89%) the method detecting DM was 18F-FDG PET-CT; in one case it was CT. The VL was significantly higher in patients who developed DM than in those who were cured (VL 4.09, *n* = 9 vs. VL 3.25, *n* = 76; *p* = 0.009), or when compared to all the others (VL 4.09, *n* = 9 vs. VL 3.24, *n* = 82; *p* = 0.014; Table 1). The most common site of DM was the lungs (56%, *n* = 5, VL 4.24 ± 0.5). Other DM were found in the cerebellum (22%, *n* = 2, VL 3.82 ± 0.4), liver (11%, *n* = 1; VL 3.16), or in more than one organ simultaneously (11%, *n* = 1; VL 4.76). There were 0, 4, 2, and 3 patients with DM in the N0, N1, N2, and N3 groups, respectively (*p* = 0.930). In the patients with T1, T2, T3, and T4, 0, 3, 2, and 4 DM appeared, respectively (*p* = 0.593). 

**Figure 3:**
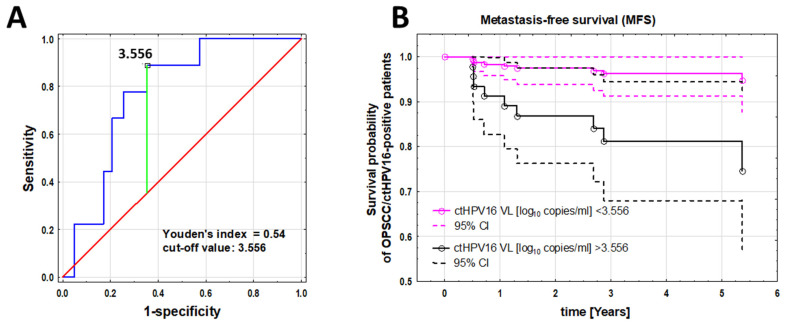
(**A**). ROC curve showing the VL cutoff of 3.556 for predicting DM (AUC 0.774, *p* < 0000); blue, pretreatment VL (log_10_ copies/mL), green, Youden index; red, random classifier. (**B**). MFS probability of OPSCCC patients depending on the cutoff value. ctHPV16 VL (log_10_ copies/mL) > 3.556 as a significant independent prognostic factor for the occurrence of DM (HR 5.5; 95% CI 1.14–26.5, *p* = 0.033). Abbreviations: DM—distant metastasis, VL—viral load, ROC—receiver operating characteristic, AUC—area under the ROC curve, MFS—metastasis-free survival, ctHPV16, circulating tumor-related human papillomavirus type 16; OPSCC, oropharyngeal squamous cell carcinoma.

The ROC curve in Figure 3A revealed a cutoff value of 3.556 for DM prediction (AUC 0.774, *p* = 0.00001). More DM appeared in the patients with a VL > 3.556 than in the patients with a VL < 3.556 (7 v 2, *p* = 0.013). The sensitivity of patients with a VL above the cutoff value who developed DM was 78%. The specificity was estimated at 65%, and the negative and positive predictive values were 96% and 19%, respectively.

For patients with a ctHPV16 VL > 3.556, the 2-, 3-, and 5-year MFS rates were 87%, 81%, and 81%, respectively. For patients with a VL < 3.556, the 2-, 3-, and 5-year MFS rates were 97%, 96%, and 96%, respectively. Finally, for MFS, we performed a Cox proportional hazards regression analysis that included a cutoff value of 3.556 and the following parameters: T3/T4 vs. T1/T2, N3 vs. N0–2, sex, smoking, and age < 59 vs. age ≥ 59 years. Backward analysis showed that a VL > 3.556 was a significant independent prognostic factor for DM (HR 5.5; 95% CI 1.14–26.5, *p* = 0.033, Figure 3B). For primary tumor localization in the tonsil, in the patients with a VL > 3.556 the risk of DM was 5.5 (*p* = 0.033), maintaining its status as an independent predictor.

## 4. Discussion

To our knowledge, this is the first study to demonstrate that ctHPV16 DNA VL assessed in liquid biopsy has prognostic value of DM risk in patients with HPV-related OPSCC. It should be emphasized that our research was based on a 10-year follow-up. We found that an increase in the VL by 1 log increased the risk of DM two times. The VL cutoff value was calculated at 3.556. In this study, we showed that in the group of patients with a VL > 3.556 the risk of DM was 5.5 higher compared to the patients with a VL < 3.556. Because 18F-FDG PET-CT was performed only in 53% of the patients (48 patients prior treatment), the potential risk of occult DM at the time of diagnosis could not be excluded. Because DM occurred mostly within 16 months of treatment completion (67% of all DM), it could not be ruled out that there were micrometastases already at the time of diagnosis. Thus, ctHPV16 VL may be useful for indication of potentially metastatic disease before treatment. Namely, regardless of T or N status, high VL may indicate a high risk of metastasis and therefore more advanced disease that requires more aggressive treatment. Similar to our study, Hanna et al. showed a trend of higher VL in patients with lung metastases, and showed that the locoregional disease had lower VL versus pulmonary-only metastases [16]. However, there is only one report which indicated high VL of ctHPV DNA as an independent predictor of DM, but it was performed on oral cavity squamous cell carcinoma [17].

At the same time, it should be noted that among the patients with a high VL, there were still patients who did not relapse. The calculated positive predictive value, which reflected the proportion of patients with a VL > 3.556 who developed DM, was low (19%). This indicates that a large group of patients with a high VL (>3.556) did not develop DM. This problem was highlighted by the high HR (4.23) of the upper CI for MFS (Table 3) as well as the 94% 5-year MFS for the upper confidence interval of the group with a VL above 3.556 (Figure 3B). These results confirmed the existence of patients with an initial high VL and good prognosis. To understand the implications of VL on survival, the issue of VL should be discussed in more detail based on studies using HPV testing in tumor tissues. The advantage of HPV detection over point mutation detection in tumors is that there are multiple copies of HPV DNA in a tumor cell. The predominant ratio of HPV DNA copies to control gene copies results in the predominant number of HPV DNA copies released during the degradation of a single cancer cell. Indirect evidence of this hypothesis is the positive correlation between the number of HPV copies in the tumor and the number of ctHPV copies in the blood [15,18]. It should therefore be noted that high VL may be due to a very high copy number per cell and not the tumor stage, and such patients may not experience recurrence or DM.

An interesting issue in our research was the positive correlation between VL and N status, with particularly high VL in the patients with N3 status. Similar positive correlations between N status and high VL were observed by Veyer et al. [19]. Chera et al. observed significantly higher ctHPV16 levels in patients with N2a/N2b compared with N0/N1 (AJCC 7th edition) and a trend toward lower VLs in patients with N2c than in those with N2a/N2b [20]. N status may result from the increased proliferative potential induced by high VL per cell or it may be caused by the stage of the disease. The lack of subepithelial layers of connective tissue under the tonsillar crypts may be the reason for early lymph node involvement. Before the primary lesion grows, cancer cells from the basal cell layer move to the lymph nodes in the neck. In this way, the primary lesion and metastases in the lymph nodes grow simultaneously [21]. Because the HPV increases the proliferative potential, the tumor growth process is enhanced and perhaps even stronger in the lymph nodes. Whether high viral load enhances this process has not been investigated. Indirect evidence can be found in Cohen’s work, in which the probability of recurrence was statistically lower as the HPV viral load per tumor cell increased. They also observed a significant increase in the overall survival probability as the viral load per tumor cell increased [22]. So, it is possible that high VL per cell increases the proliferative potential of the primary tumor and metastases in the lymph nodes. It can therefore be concluded that high HPV VL in this particular type of OPSCC cancer leads to a pseudo-advanced stage of the disease, which in turn has a better prognosis. Also plausible is the hypothesis put forward by Cohen et al. that residual or recurrent microscopic tumor cells with higher viral loads may be more easily attacked by HPV-specific T cells [22].

Studies using tumor tissue for measuring VL indicate that high VL is a good prognostic factor for OS in HNC patients [23,24]. In patients with the highest VLs of HPV16-associated OPSCC located in the tonsil, OS and DFS (disease-free survival) improvements were also observed [22,24]. Another study showed that the HPV VL was significantly higher in cancer samples than in nonmalignant samples [25]. However, there are also several reports showing that patients with low VL in the tumor have a worse survival [22,23,24,26]. Hashida et al. reported significantly worse OS and progression-free survival (PFS) in p16-positive OPSCC patients with no HPV DNA or low tissue VL than in patients with high VLs [24]. In other studies, good concordance between the presence of viral transcripts and high VL was observed for identifying HPV16-related tumors in OPSCC patients [23]. In contrast, in one study, neither VL nor HPV16 DNA integration was associated with OS or PFS [27]. In our research, the plasma-based VL was not related to OS or LRFS (*p* = 0.70 for both, Table 3). However, Hanna et al. showed that patients with a lower overall tumor burden demonstrated lower median plasma VL which corresponded with improved survival [16]. Despite similar 2-year OS, the 5-year OS was much lower in their study than in ours (66.7% compared to our value of 81%). Such differences may be due to the use of qPCR to classify patients into a plasma-based ctHPV-dependent group [15]. Because qPCR is less sensitive than digital droplet (ddPCR), we may not have been able to detect low copy numbers, so the patients with a low number of copies in the tumor had a negative result in the blood. The effect of this underestimation was the exclusion of the patients with a low VL in the tumor, which may have resulted in a higher OS in our group. It is therefore possible that the increased 5-year OS rate in our studies was due to the failure to detect a low copy number. Low copy number in tissue is one of the factors contributing to the inability to detect ctHPV16 and patients with HPV-positive OPSCC. As we have shown in our previous studies, the most important factors contributing to the lack of detection of cHPV16 DNA in OPSCC patients were low viral load in tissue and low N stage [15]. Survival analyses in the context of liquid biopsy-based VL are yet to be conducted.

If our findings are confirmed, VL in liquid biopsy may become a stratification factor for optimal treatment selection. Some authors suggested that patients treated with induction chemotherapy had a lower rate of DM than others. However, no difference in OS, disease-free survival, or locoregional recurrence was observed in these cohorts [28,29]. Our research did not confirm this, as we did not find a significant difference in the occurrence of DM in the iCHT treatment regimen compared to that in the concurrent CHRT regimen (OR 2.67, *p* = 0.371). Currently, a number of studies are being conducted on the immunophenotypes in HPV-related cancers. It should be emphasized that both PD-1 and PD-L1 expression on immune cells are favorable prognostic biomarkers in patients with HPV-related OPSCC [30,31]. Recently, immune checkpoint inhibitors such as pembrolizumab and nivolumab have been approved for the treatment of patients with recurrent or metastatic disease [32].

In this study, we did not estimate the correlation between metabolic tumor burden determined with 18F-FDG PET-CT and pretreatment shedding of circulating tumor DNA. However, Tatsumi et al. demonstrated that ctHPV16 levels correlated with the whole-body tumor burden visualized by 18F-FDG PET-CT. They suggested that ctHPV16 DNA and PET-CT may complement each other, which would be useful in post-treatment management decisions [33]. Several studies have highlighted a positive correlation between high metabolic tumor burden and ctDNA detection in patients with locally advanced, previously untreated HNSCC [33,34,35]. In the era of precision oncology, multiparametric tests will be helpful in creating prognostic and predictive algorithms in the management of patients with ctHPV-positive HNSCC [34].

The methodology for detecting unique HPV DNA sequences in liquid biopsy as tumor identifiers is diverse. Due to the relatively low cost, simplicity, and availability of equipment in most laboratories, qPCR and ddPCR are the most frequently used [36,37]. A common feature of qPCR and ddPCR is the use of the set of fluorescent probe and primers to detect a specific fragment (target fragment), which can be used interchangeably in these methods. ddPCR is designed stoichiometrically so that there is only one template per droplet, thus giving a chance in the case of a low copy number that one of the droplets will contain the target fragment and it will be amplified enough for fluorescence reading (by the detector), with simultaneous amplification of the control gene. In the case of qPCR, the stoichiometric ratio is not taken into account and one reaction volume may contain many control gene templates and a very small number of the target fragment. During the reaction, accumulation of too much control product may inhibit amplification of the target gene. Therefore, qPCR is a less sensitive technique [38]. Recently, next-generation sequencing (NGS) technology was presented as the best option, as it allows the detection of many types of HPV during one reaction, in addition to detecting low copy numbers [39,40]. Another research paper compared the use of qPCR, ddPCR, and NGS for the detection of ctHPV in liquid biopsy of various cancers, including oral and cervical cancer [41]. Pooled specificity for ctHPV was similar for qPCR, ddPCR, and NGS, while pooled sensitivity was highest for NGS, followed by ddPCR, and then qPCR. More interestingly, when sensitivity was compared between anatomical locations across all the platforms, detection by qPCR or ddPCR was better in the oropharynx compared to the cervix (the sensitivity for ddPCR was better compared to qPCR). However, NGS significantly improved the sensitivity for cervical cancer, while the sensitivity for OPSCC was comparable between NGS and ddPCR. Therefore, the detection platform and anatomical location of the tumor should be taken into account when interpreting ctHPV test results [41].

The proposed classification for HPV-related OPSCC, which was presented by The International Collaboration on Oropharyngeal Cancer Network for Staging (ICON-S) in 2017, permitted a more appropriate depiction of prognosis than the 7th edition [5,42]. Interestingly, in our previous study conducted in 2019 we did not reveal a correlation between VL and N status (or with T status), however, the 7th edition of the AJCC was used then [15]. In the current study, which used the 8th edition of the AJCC, we showed a correlation between ctHPV16 copy number and N status (whereas no correlation with T status was still maintained). The new N classification in the 8th edition of the AJCC appears to be more aligned with VL. Therefore, given the possibilities of liquid biopsy-based diagnostics, hard evidence is needed to determine the suitability of ctHPV as an adjunct or alternative that will benefit patients when used in routine diagnostics [43].

## 5. Conclusions

Our results suggest that a high initial VL in ctHPV-positive OPSCC may indicate a higher risk of DM. VL, not N or T status, should be considered as a stratifying parameter for treatment selection in the future. Further research on the implementation of ctHPV is urgently needed to improve this biomarker for further personalization of frontline clinical treatments.

## Figures and Tables

**Table 1:** Comparative analysis of VL according to clinical parameters of patients with ctHPV16-related OPSCC.

	A	B	C	D	E
Parameter	*n* (%)	ctHPV16 (Copies/mL) Median (l.q.–u.q.)	ctHPV16 VL (log_10_ Copies/mL) Median (l.q.–u.q.)	ctHPV16 VL (log_10_ Copies/mL) Mean (±SD)	*p*-Value
	91	2367 (424–11,580)	3.37 (2.63–4.06)	3.32 ± 0.99	
Sex					
female	32 (35)	868 (159–4960)	2.9 (2.2–3.7)	3.03 ± 1.09	0.052
male	59 (65)	2840 (908–13,700)	3.5 (3.0–4.1)	3.48 ± 0.90	
Age (years, median)					
<59	44 (48)	1847 (408–15,912)	3.27 (2.61–4.20)	3.36 ± 1.09	0.752
≥59	47 (52)	2680 (704–7914)	3.43 (2.85–3.90)	3.29 ± 0.90	
T classification					
1	3 (3)	2900 (156–29,000)	3.46 (2.19–5.46)	3.71 ± 1.65	
2	36 (40)	1037 (283–10,663)	3.01 (2.39–4.01)	3.17 ± 1.02	0.355
3	28 (31)	2306 (844–3998)	3.36 (2.93–3.60)	3.21 ± 0.83	(T3/T4 vs. T1/T2)
4	24 (26)	5924 (952–13,700)	3.77 (2.89–4.14)	3.64 ± 1.01	
N classification					
0	2 (2)	516 (328–704)	2.68 (2.52–2.85)		
1	46 (51)	1509 (179–7724)	3.18 (2.25–3.89)	3.22 ± 1.03	**0.022**
2	18 (20)	2071 (182–6247)	3.30 (2.26–3.80)	3.13 ± 1.01	**(N3 vs. N0–2)**
3	25 (27)	3916 (1635–13,880)	3.59 (3.21–4.14)	3.71 ± 0.84	
cigarette consumption					
non-smoker	51 (56)	1635 (182–11,580)	3.21 (2.26–4.06)	3.28 ± 1.00	0.609
smoker	40 (44)	2770 (887–9284)	3.44 (2.95–3.94)	3.38 ± 0.98	
localization					
tonsil	71 (78)	2680 (424–12,246)	3.43 (2.63–4.09)	3.35 ± 0.96	0.310
base of tongue	19 (21)	1148 (156–3820)	3.06 (2.19–3.58)	3.10 ± 0.90	(tonsil vs. base of tongue)
palate	1 (1)	976,145 (n/a)	5.99 (n/a)		
according LRR					
without LRR	81	1964 (424–10,523)	3.29 (2.63–4.02)	3.31 ± 1.01	0.599
with LRR	10	4418 (804–12,246)	3.64 (2.91–4.09)	3.48 ± 0.83	
according DM					
without DM	82	1683 (328–7724)	3.23 (2.52–3.89)	3.24 ± 0.99	**0.014**
with DM	9	12,246 (6247–13,742)	4.09 (3.80–4.14)	4.09 ± 0.58	
tonsil cancer only					
without LRR	61	2367 (424–11,580)	3.37 (2.63–4.06)	3.33 ± 0.99	0.642
with LRR	10	4418 (804–12,246)	3.64 (2.91–4.09)	3.48 ± 0.83	
tonsil cancer only					
without DM	62	1847 (328–7914)	3.27 (2.52–3.90)	3.24 ± 0.96	**0.012**
with DM	9	12,246 (6247–13,742)	4.09 (3.80–4.14)	4.09 ± 0.58	

l.q., lower quartile; u.q., upper quartile; SD, standard deviation; DM, distant metastasis; LRR, locoregional recurrence.

**Table 2:** Locoregional recurrence-free survival (LRFS), metastasis-free survival (MFS), and overall survival (OS) rates of ctHPV16-related OPSCC patients.

	LRFS	MFS	OS
2 years	92%	93%	94%
3 years	89%	90%	92%
5 years	88%	90%	81%
9 years	88%	86%	66%

**Table 3:** Multivariate Cox’s proportional hazards regression models for LRFS, MFS, and OS of ctHPV16-related OPSCC patients treated with RT/CHRT.

	LRFS				MFS				OS			
PARAMETER	Full Model (All Effect)		Backward Elimination *		Full Model (All Effect)		Backward Elimination *		Full Model (All Effect)		Backward Elimination *	
	HR (95% CI)	*p* Value	HR (95% CI)	*p* Value	HR (95% CI)	*p* Value	HR (95% CI)	*p* Value	HR (95% CI)	*p* Value	HR (95% CI)	*p* Value
Age at diagnosis (ref <59)	0.85 (0.22−3.19)	0.807			2.42 (0.54−10.80)	0.391			0.82 (0.27−2.49)	0.728		
Male vs. female (ref. female)	0.61 (0.16−2.35)	0.473			3.47 (0.42−28.93)	0.251			1.02 (0.31−3.33)	0.980		
Smoker vs. non-smoker (ref. non-smoker)	1.73 (0.46−6.45)	0.417			1.00 (0.22−4.55)	0.996			1.10 (0.36−3.38)	0.863		
T3/T4 vs. T1/T2 status (ref. T1/T2)	8.12 (0.99−66.83)	0.052	7.27 (0.92−57.44)	0.060	1.10 (0.26−4.70)	0.896			1.53 (0.45−5.24)	0.501		
N3 vs. N0−N2 status (ref. N0−2)	0.27 (0.03−2.35)	0.233			1.16 (0.24−5.71)	0.851			0.52 (0.13−2.14)	0.363		
ctHPV16 VL (log_10_ copies/mL) (cont.)	1.12 (0.63−2.00)	0.705			2.31 (1.10−4.87)	0.027	2.22 (1.17−4.23)	**0.015**	1.12 (0.64−1.96)	0.700		

*—All parameters were taken for backward elimination; LRFS, locoregional recurrence-free survival; MFS, metastasis-free survival; OS, overall survival; ctHPV16, circulating tumor-related human papillomavirus type 16; OPSCC, oropharyngeal squamous cell carcinoma, RT/CHRT, radiotherapy or chemoradiotherapy (ref.)—indicates a reference parameter, (cont.): continuous variable; HR: hazard ratio; CI: confidence level.

## Data Availability

The datasets used and/or analyzed during the current study are available from the corresponding author upon reasonable request.

## Abbreviations

AUC—area under the curve; CHRT—concurrent chemoradiotherapy; ctHPV16—circulating tumor-related human papillomavirus type 16; DM—distant metastases; HPV—human papillomavirus; HR—hazard ratio; iCHT—induction chemotherapy; LRFS—locoregional recurrence-free survival; MFS—metastasis-free survival; OPSCC—Oropharyngeal Squamous Cell Carcinoma; OS—overall survival; qPCR—quantitative polymerase chain reaction; ROC—receiver operating characteristic; RT—radiotherapy; VL—viral load.
